# Acute elevation of intra-abdominal pressure contributes to extravascular shift of fluid and proteins in an experimental porcine model

**DOI:** 10.1186/1756-0500-7-738

**Published:** 2014-10-20

**Authors:** Bjørg Elvevoll, Paul Husby, Kjell Øvrebø, Oddbjørn Haugen

**Affiliations:** Department of Anesthesia and Intensive Care, Haukeland University Hospital and University of Bergen, N-5021 Bergen, Norway; Department of Surgery, Haukeland University Hospital and University of Bergen, N-5021 Bergen, Norway

**Keywords:** Intra-abdominal hypertension, Fluid extravasation, Protein extravasation, Hypo-perfusion, Helium

## Abstract

**Background:**

Intra-abdominal hypertension and abdominal compartment syndrome contribute significantly to increased morbidity and mortality in critically ill patients. This study describes pathophysiologic effects of the acutely elevated intra-abdominal pressure on microvascular fluid exchange and microcirculation. The resulting changes could contribute to development of organ dysfunction or failure.

**Methods:**

16 pigs were randomly allocated to a control-group (C-group) or an interventional group (P-group). After 60 min of stabilization, intra-abdominal pressure of the P-group animals was elevated to 15 mmHg by Helium insufflation and after 120 min to a level of 30 mmHg for two more hours. The C-group animals were observed without insufflation of gas. Laboratory and hemodynamic parameters, plasma volume, plasma colloid osmotic pressure, total tissue water content, tissue perfusion, markers of inflammation and cerebral energy metabolism were measured and net fluid balance and fluid extravasation rates calculated. Analysis of variance for repeated measurements with post-tests were used to evaluate the results with respect to differences within or between the groups.

**Results:**

In the C-group hematocrit, net fluid balance, plasma volume and the fluid extravasation rate remained essentially unchanged throughout the study as opposed to the increase in hematocrit (P < 0.001), fluid extravasation rate (P < 0.05) and decrease in plasma volume (P < 0.001) of the P-group. Hemodynamic parameters remained stable or were slightly elevated in the C-group while the P-group demonstrated an increase in femoral venous pressure (P < 0.001), right atrial pressure (P < 0.001), pulmonary capillary wedge pressure (P < 0.01) and mean pulmonary arterial pressure (P < 0.001). The protein mass decreased in both study groups but was significantly lower in the P-group as compared with the C-group, after 240 min of intervention. The increased intra-abdominal pressure was associated with elevated intracranial pressure and reduced tissue perfusion of the pancreas and the gastric- and intestinal mucosa.

**Conclusion:**

Elevation of intra-abdominal pressure has an immediate impact on microvascular fluid extravasation leading to plasma volume contraction, reduced cardiac output and deranged perfusion of abdominal organs.

## Background

Intra-abdominal hypertension (IAH) and abdominal compartment syndrome (ACS) are known to affect morbidity and mortality in the critically ill patient [1, 2]. Depending on the patient populations, the incidence of IAH has been reported in the range of 32-50% [2–4].

The occurrence of ACS also varies among specific groups of patients, with an incidence found to be around 4% in a general medical-surgical population [2, 5].

The underlying pathophysiological mechanisms are not fully understood, but are frequently related to an increase in the intra-abdominal volume resulting in a shift to the steeper part of the abdominal pressure-volume curve. A reduction in abdominal wall compliance could lead to the same result. Factors contributing to an increase in intra-abdominal volume could be regional such as intra-abdominal hemorrhage, ascites formation or intestinal dilatation [6, 7]. The condition may also follow massive fluid resuscitation, as in sepsis or burn injuries, leading to increased extravasation of fluid from the vascular to the interstitial space and edema formation [8].

The transcapillary fluid shift is basically determined by the forces of the classic Starling equation containing the capillary filtration coefficient, the reflection coefficient for plasma proteins, and the balance between the intra- and extra-capillary hydrostatic and colloid osmotic pressures [9]. The capillary hydrostatic pressure (Pc) is influenced partly by the pre- and post-capillary resistance, but more strongly by the central venous pressure. Previous studies on intra-abdominal hypertension have demonstrated that this condition may be accompanied by elevated central venous pressure [10].

In the present study we assessed the early changes in fluid- and protein shifts following acute elevation of intra-abdominal pressure (IAP) to a level of 15 and thereafter 30 mmHg, corresponding to grade 2 and grade 4 of IAH, respectively [7]. Based on previous observations on central venous pressure during IAH, we hypothesized that acute elevation of intra-abdominal pressure, per se, would lead to an increase in fluid filtration from the capillaries to the interstitial space.

## Methods

### Animal handling and anesthesia

16 immature domestic pigs (Norwegian landrace-Yorkshire hybrid) were studied. The animal handling was following recommendations given by the Norwegian State Commission for Laboratory animals and in accordance with internationally recognized guidelines. The study protocol was approved by the Institutional Veterinarian Authority (12.03.2012 # 20124218) prior to start of the study. All animals were acclimatized in the facilities for at least five days before the experiments. Water was provided at all times, but food was withdrawn 8-12 h prior to induction of anesthesia.

The animals were pre-medicated with Ketamine 500 mg, diazepam 10 mg and atropine 1 mg i.m. General anesthesia was induced via a face mask with Isoflurane in oxygen, supplemented by Thiopentone 5 mg/kg i.v. after establishing i.v. access in an ear vein. They were then oro-tracheally intubated (Mallinckrodt, blueline oral tube, I.D 6.0, Covidien plc, Dublin, Ireland). General anesthesia was maintained by volume-controlled ventilation with Isoflurane 0.5-2.0% delivered in 40-60% oxygen in air via a ventilator (Julian anesthesia work station, Dräger, Lübeck, Germany) and continuous i.v. infusion of Midazolam (0.5 mg/kg/h) and Fentanyl (7.5 μg/kg/h). The anesthesia protocol is previously described [11].

All animals were given continuous i.v. infusion of acetated Ringer’s solution (10 ml/kg/h) from induction of anesthesia. Blood samples and bleeding were substituted for by acetated Ringer’s solution in volumes three times the volume of blood lost from circulation. The amount of blood loss was measured by weighing the surgical sponges. Hypothermia was prevented by heating blankets. At the end of the experiments, the animals were killed by an i.v. injection of 20 ml saturated KCl-solution.

### Surgical procedures and hemodynamic monitoring

A cranial burr hole with a diameter of 0.5 cm was made in the frontal bone of the pig skull while in the prone position. Dura mater was incised and a pressure transducer (Codman MicroSensor ICP Transducer, Johnson & Johnson Proffesional Inc., Raynham, MA, USA) introduced 20 mm into the brain parenchyma and connected to a Codman Express™ monitor. A microdialysis catheter (CMA 70, 20 mm, Cutoff 20 000 Dalton, CMA microdialysis AB, Stockholm, Sweden) was inserted through the burr hole and placed with the tip 25 mm into the brain parenchyma.

Thereafter, in the supine position, the femoral artery and vein were surgically exposed and catheters introduced into the respective vessels (Secalone T™, 18G, BD Medical, Singapore). Mean arterial pressure (MAP) and femoral venous pressure (CVP_FV_) were continuously monitored through fluid-filled lines connected to transducers (Transpac^TM^ П, Abbot Critical Care Systems, Sligo, Ireland) linked to a monitor (HP-78353A, Hewlett Packard, Palo Alto, CA, USA). Heart rate was monitored through surface ECG-electrodes.

The right external jugular vein was surgically exposed and a Swan-Ganz catheter introduced and positioned in the pulmonary artery for recording of mean pulmonary artery pressure (MPAP), pulmonary capillary wedge pressure (PCWP), right atrial pressure (CVP_RA_) and continuous cardiac output (CCO) (7,5 F Swan Ganz Thermodilution catheter, Edwards Lifesciences, Irvine, CA, USA). The pressure values were displayed on the same monitor as were femoral arterial- and venous pressure while CCO was processed and displayed by a Vigilance II unit (Edwards Lifesciences, Irvine, CA, USA). The left carotid artery was surgically exposed and a single lumen polyethylene catheter placed with the tip in the left ventricle, for injection of fluorescent microspheres. The position was verified by pressure curve assessment.

Finally a mini-laparotomy was performed through an incision to the left of the abdominal midline above the pubic bone. The urinary bladder was punctured and a Foley catheter introduced for monitoring of urinary output. The abdominal wall was punctured and a triple lumen central venous catheter (12 F 3-lumen catheter, 16 cm, Arrow-Howes™ Large-Bore Multi-lumen CVC-Set, Arrow International, Inc. Reading, PA, USA) was placed in the abdominal cavity, with the tip in the left upper quadrant. The catheter was linked to a pressure transducer for monitoring of IAP and to a 10 liter cylinder of Helium (≥99,996% Helium, Aga AS, Oslo, Norway) for insufflation of gas. To obtain an airtight closure of the abdominal wall, a thin plastic film was placed over the bowels before the wound was closed in layers by running stiches.

### Study protocol

The animals were randomized to either the intervention group (P-group, n = 8) or the control group (C-group, n = 8). Following surgical preparation, all animals were stabilized for 60 minutes before intervention. The intra-abdominal pressure of the P-group was then elevated to 15 mmHg through insufflation of Helium into the abdominal cavity. After 120 minutes the pressure was further elevated to 30 mmHg and maintained at this level for 120 minutes. When necessary, additional helium was insufflated to maintain the target level of pressure. The C-group animals were observed for the same time period without interventions.

### Plasma-volume, net fluid balance and fluid extravasation rate

After 30 minutes of stabilization, red blood cell volume (V_RBC_) was determined using carbon monoxide as label. The method is previously presented in detail [12]. During the rest of the experiment new values for V_RBC_ were calculated every 30 minutes based on baseline V_RBC_, repeated hematocrit measurements and recorded loss of blood. The corresponding values for plasma volume (PV) were also calculated. Urine output was recorded every 30 minutes, and net fluid balance (NFB) was calculated as the total amount of fluid added minus the recorded loss of fluid during every 30 minutes. Fluid extravasation (FE) was defined as NFB minus the change in PV (ΔPV) over the same time interval:


The resulting fluid extravasation rate (FER) was presented as ml/kg/min.

### Blood chemistry

Blood samples were drawn from the arterial line for determination of hemoglobin concentration (Hb), hematocrit (Hct), serum-albumin, serum-total protein, serum-electrolytes, serum-osmolality and acid-base parameters. Hct was determined using standard hematocrit-tubes centrifuged at 12000 rpm for 10 min. Serum-albumin, serum-total protein concentrations and serum-electrolytes were analyzed in an automatic analyzer (Clinical analyzer 7600, Modular P, Hitatchi High-Tecnologies Corporation, Tokyo, Japan). Albumin- and protein masses were calculated by multiplying serum levels with simultaneous plasma volume. Serum-osmolality was analyzed by freezing point determination. Blood glucose was analyzed by test strips (MediSense precision Xtra glucose analyzer, MediSense UK, Oxon UK).

### Microdialysis

The microdialysis catheter was perfused with CNS perfusion fluid (CMA) by a microinfusion pump (CMA 107) with a flow rate of 0.3 μl/min. Samples of microdialysate were collected in microvials and analyzed by a CMA 600 Microanalyser every 30 minutes with respect to glucose, lactate, pyruvate and glycerol. The method is previously validated [13].

### Inflammatory mediators

Serum levels of tumor necrosis factor alpha (TNF-*α*) and interleukine-6 ( IL-6) were measured by a quantitative sandwich enzyme immunoassay technique (Quantikine, Porcine Immunoassay by R&D Systems, Minneapolis, MN, USA) according to the manufacturer protocol. The reported mean minimum detectable dose of IL-6 and TNF-*α* were 2.03 pg/ml and 3.7 pg/ml, respectively.

### The microsphere method

Regional blood flow was measured by colored microspheres in different tissues and organs. The method is based upon the notion that 15 μm colored spheres after homogenous mixing in arterial blood become trapped in the capillaries to an extent proportional to the regional blood flow [14]. Microspheres were infused into the left ventricle through the catheter in the left carotid artery followed by constant rate retraction of blood from the femoral artery for reference. The number of spheres in various tissues and reference blood was calculated by digestion of samples in 4 Molar KOH and subsequent filtration through a 10 μm Teflon filter. The dye was released from the spheres with dimethylformamid and the content of each dye was then measured in a spectrophotometer.

### Total tissue water (TTW)

Immediately after killing the animal, tissue samples, in doubles, were taken from left quadriceps muscle, right- and left ventricular myocardium, lung, liver, left- and right kidney, the mucosal-and muscular layer of the ventricle, pancreas, the mucosal and muscular layer of the ileum, brain and abdominal skin. The tissue samples were placed in pre-weighed vials, reweighed and placed in a drying chamber at 70°C. The vials were weighed repeatedly until stable weight, and the weight reduction was considered to represent the water content of the tissue. TTW was recorded as g/g dry weight.

### Statistical analysis

All data were analyzed with IBM SPSS 21 for Windows (SPSS Inc., Chicago, IL, USA). Graphic presentations were made by use of GraphPad Prism Ver 5.01 (GraphPad Software, Inc, La Jolla, CA, USA). Analysis of variance for repeated measurements, with one within group factor (time) and one between group factor (intra-abdominal pressure) was used. On finding a significant between-group difference or a significant interaction between the study groups, independent t-tests or Mann Whitney U test were performed to compare the groups at baseline and after 60, 120, 180 and 240 minutes of intervention. If a significant within-group difference was found, paired t-tests were performed to compare the baseline values with the values obtained after 60, 120, 180 and 240 minutes of intervention.

For hematocrit, plasma volume, net fluid balance and fluid extravasation rate the statistical analysis were based upon average values during the 60 minutes of stabilization (Baseline), the first 120 minutes, and the last 120 minutes of the intervention.

## Results

The animals of the C-group and P-group were comparable with respect to age: 103.9 (22.3) and 93.1 (10.3) days, weight: 41.94 (2.65) and 41.92 (3.1) kg and surface area: 1.1 (0.04) and 1.1 (0.05) m^2^, respectively. The gender of the C-group and P-group animals were 7/1 and 3/5 (male/female) in the respective groups.

**Figure 1:**
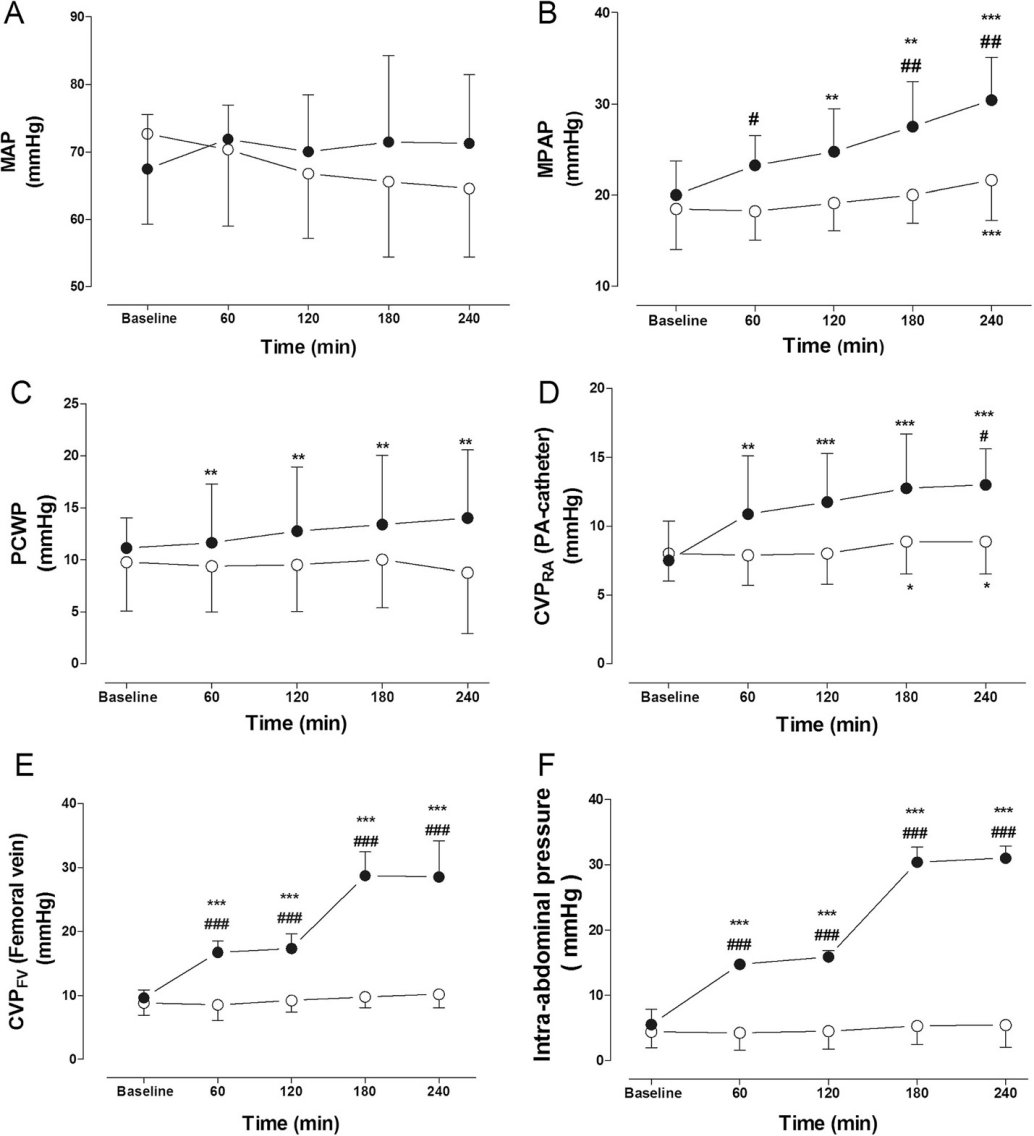
**The figure shows A: Mean arterial pressure (MAP), B: Mean pulmonary arterial pressure (MPAP), C: Pulmonary capillary wedge pressure (PCWP), D: Right atrium central venous pressure (CVP**_**RA**_ **), E: Femoral vein central venous pressure (CVP**_**FV**_ ) and F: Intra-abdominal pressure (IAP) in the ●: Intervention group (P-group) and the ○: Control-group (C-group) **: P < 0.01 ***: P < 0.001 within group difference compared with baseline values; # : P < 0.05 ##:P < 0.01 ###: P < 0.001 between group difference, same time.

The Baseline values in the tables and figures correspond to measured or calculated values after 1 hour of stabilization. Mean arterial pressure (MAP), mean pulmonary artery pressure (MPAP), pulmonary capillary wedge pressure (PCWP), central venous pressure obtained from the right atrium (CVP_RA_) and the femoral vein (CVP_FV_) and intra-abdominal pressure (IAP) are displayed in Figure 1. MAP remained essentially stable with no between-group differences during the study. In the P-group MPAP increased from 20.0 (3.7) mmHg at baseline to 30.4 (4.7) mmHg after 240 min (P < 0.001) in contrast to the modest changes in the C-group with 18.5 (4.5) mmHg at baseline and 21.6 (4.4) mmHg after 240 min (P < 0.001). Between-group differences were present from 60 minutes after intervention (P < 0.05). Also, PCWP showed an increase in the P-group from 11.0 (3.0) mmHg at baseline to 16.0 (3.6) mmHg at the end of the experiment. In the C-group PCWP remained stable. CVP_RA_ increased slightly in the C-group with 8.0 (2.0) mmHg at baseline and 8.9 (2.4) mmHg at 240 min (P < 0.05) in contrast to the P-group where corresponding values increased from 7.5 (2.9) mmHg to 13.0 (2.6) mmHg (P < 0.001 within-group and P < 0.05 between-group differences at 240 min). A similar, but more obvious pattern was seen in CVP_FV,_ where the values of the C-group were 8.8 (1.9) mmHg at baseline and 10.2 (2.1) mmHg after 240 min while CVP_FV_ of the P-group increased from 9.6 (1.3) mmHg at baseline to 17.3 (2.3) mmHg after 120 min (P < 0.001) and 28.5 (5.7) mmHg after 240 min (P < 0.001). The values differed significantly between the groups from 60 minutes after intervention (P < 0.001). They closely followed IAP which in the C-group was 4.4 (2.4) mmHg at baseline and 5.4 (3.4) mmHg at the end of the experiment, and in the P-group was 5.5 (2.4) mmHg at baseline and finally 30.0 (1.9) mmHg.

The results of cardiac output (CO), systemic vascular resistance (SVR), pulmonary vascular resistance (PVR), intracerebral pressure (ICP) and cerebral perfusion pressure (CPP) are displayed in Table 1. CO, SVR and PVR remained essentially stable in the C-group throughout the experiment. In the P-group CO decreased significantly at 180 min (P < 0.05) and 240 min (P < 0.05) as compared with baseline values and after 180 min CO was significantly lower than the corresponding value of the C-group (P < 0.05). As opposed to the C-group, SVR increased in the P-group and differed significantly from the C-group after 180 min of intervention (P < 0.001 within-group-, P < 0.05 between-group differences). PVR of the P-group was significantly elevated after 240 min when compared to baseline (P < 0.05) and a tendency to increasing values of PVR was also seen in the C-group. ICP increased significantly in the P-group after 120 min (P < 0.001). No significant increase in ICP of the C-group was found although a trend was seen. With respect to CPP, a slight decreasing trend was present in both groups during the experiments with no between-group differences.

**Figure 2:**
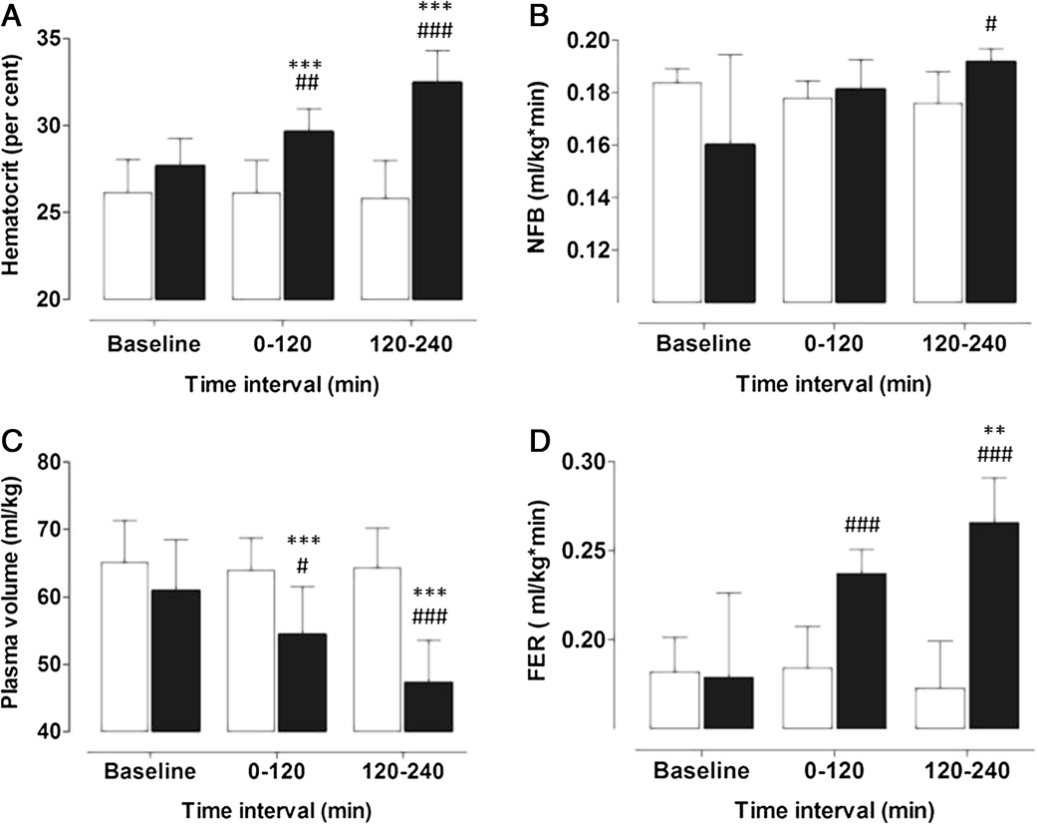
The figure displays A: Hematocrit, B: Net fluid balance, C: Plasma volume and D: Fluid extravasation rate in the Intervention group (P-group) (black columns) and the control group (C-group) (white columns); **: P < 0.01 ***: P < 0.001 within group difference compared with baseline values; #: P < 0.05 ##: P < 0.01 ###: P < 0.001 between group difference, same time.

Figure 2 displays the average levels of hematocrit, net fluid balance (NFB), plasma volume (PV) and fluid extravasation rate (FER) at baseline, during the first 120 min (0-120 min) and the last 120 min (120 to 240 min) of the experiments in the respective study groups. In the P-group, hematocrit increased from 27.7 (1.6)% at baseline to 32.4 (1.9)% at 120 to 240 min (P < 0.001) while the values of the C-group remained stable at 26.1 (1.9) and 25.8 (2.2)%. PV of the P-group was reduced from 60.9 (7.5) ml/kg at baseline to 47.2 (6.3) ml/kg (P < 0.001) at 120 to 240 min. The corresponding values of the C-group were 65.1 (6.2) ml/kg and 64.3 (5.9) ml/kg. A trend to increasing NFB was seen in the P-group with 0.16 (0.03) ml/kg/min at baseline and 0.19 (0.005) ml/kg/min during 120 to 240 min while the corresponding values of the C-group were 0.18(0.005) ml/kg/min and 0,18 (0.01) ml/kg/min. NFB of the study groups differed significantly at 120 to 240 min (P < 0.05). FER increased from 0.19 (0.05) ml/kg/min at baseline to 0.27 (0.03) ml/kg/min at 120 to 240 min in the P-group (P < 0.01) while the C-group values remained stable with 0.18 (0.02) ml/kg/min and 0.17 (0.03) ml/kg/min.

**Table 1:** **Hemodynamic parameters**

Parameter	Group	Baseline	60 min	120 min	180 min	240 min
**CO**	C-group	5.1 (0.6)	5.3 (0.9)	5.2 (1.1)	5.1 (0.8)#	4.8 (0.9)
(l/min)	P-group	5.4 (0.6)	5.2 (0.8)	5.1 (0.8)	3.8 (0.9)*	4.3 (0.6)*
**SVR**	C-group	905.9 (155)	876.8 (145,2)	896.4 (250,8)	835.2 (153.9)#	871.7 (134.4)
(dynes/sec/cm^-5^)	P-group	960.7 (131)	970.7 (180,5)	946.9 (120,4)	1299.2 (201.3)***	1128.8 (183.3)
**PVR**	C-group	128.6 (41.6)	123.9 (21.8)	137.8 (25.1)	144.5 (28.3)	211.9 (100.1)
(dynes/sec/cm^-5^)	P-group	134.7 (23)	138.2 (18)	151.6 (27.1)	249.4 (112.9)	250.6 (82)*
**ICP**	C-group	16.3 (2.9)	17.3 (3.1)	19.9 (4.9)	20.8 (6.0)	20.6 (5.8)
(mmHg)	P-group	16.9 (5.9)	21.6 (4.9)	24.0 (7.3)***	26.7 (4.0)***	26.9 (5.9)***
**CPP**	C-group	54.0 (11.0)	52.8 (11.7)	40.1 (20.2)	45.1 (15.2)	44.4 (13.7)
(mmHg)	P-group	52.7 (5.0)	50.9 (5.0)	48.4 (6.8)	50.6 (7.6)	47.0 (7.4)

*Values are presented as mean with SD in parentheses. Control group: C-group; Intervention group: P-group; CO: cardiac output; SVR: systemic vascular resistance; PVR: pulmonary vascular resistance; ICP: intracranial pressure; CPP: cerebral perfusion pressure.*

*: P < 0.05 ***: P < 0.001; within group difference compared with baseline values;

*#: P < 0.05; between group differences, same time.*

Laboratory parameters are presented in Table 2. The albumin and protein masses decreased in both study groups throughout the experiments although the reduction was more pronounced in the P-group. At 240 min the level protein masses was significantly lower in the P-group when compared with the C-group (P < 0.05). P_a_CO2 was significantly higher and BE lower in the P-group as compared with the C-group at 240 min resulting in a lower pH, but the numeric difference was modest. Interleukin-6 increased slightly in both study groups (P < 0.01) as did TNF-α in the P-group (P < 0.05).

**Table 2:** **Laboratory parameters**

Parameter	Group	Baseline	60 min	120 min	180 min	240 min
**Natrium**	C-group	139.4 (1.4)	139.1 (1.5)	139.1 (2.4)	139.1 (2.4)	138.2 (1.4)**
(mmol/l)	P-group	139.0 (1.5)	138.9 (1.8)	138.4 (2.3)	138.0 (3.2)	138.0 (2.1)
**Kalium**	C-group	4.2 (0.2)	4.4 (0.3)*	4.6 (0.3)**	4.9 (0.3)***	5.3 (0.5)***
(mmol/l)	P-group	4.3 (0.5)	4.5 (0.5)*	5.0 (0.7)**	5.9 (1.2)*	6.1 (0.9)***
**Chloride**	C-group	99.1 (1.4)	98.6 (0.9)	98.6 (0.9)	99.4 (1.1)	98.6 (1.1)
(mmol/l)	P-group	99.2 (1.3)	98.5 (1.2)	98.6 (2.3)	98.1 (2.0)	98.9 (1.7)
**Osmolalitet**	C-group	285.8 (2.4)	288.4 (6,6)	283.8 (1.8)	282.8 (3.6)	284.5 (4.9)
(mosm/l)	P-group	282.4 (4.6)	281.9 (2.7)	283.1 (3.6)	284.3 (3.2)	285.9 (4.7)
**s-Protein**	C-group	47.0 (2.1)	46.4 (2.1)	44.5 (2.1)**	43.4 (1.8)***	43.3 (1.2)**
(g/l)	P-group	46.9 (3.1)	49.3 (3.4)*	47.9 (3.2)	47.9 (4.4)	47.8 (3.6)#
**s-Albumin**	C-group	31.1 (4.3)	30.9 (4.0)	29.5 (4.3)**	29.8 (3.7)	28.9 (3.4)*
(g/l)	P-group	31.4 (2.9)	32.6 (3.0)	31.9 (2.4)	32.3 (2.4)	32.3 (2.7)
**Albuminmass**	C-group	2,02 (0.42)	1.98 (0.39)	1.89 (0.37)*	1.93 (0.37)	1.87 (0.41)**
(g/kg)	P-group	1.86 (0.21)	1.76 (0.22)**	1,71 (0.21)***	1.52 (0.21)***	1.45 (0.19)***
**Proteinmass**	C-group	3.03 (0.3)	2.97 (0.31)	2.84 (0.28)*	2.8 (0.25)*	2.78 (0.32)*
(g/kg)	P-group	2.79 (0.41)	2.67 (0.43)**	2.59 (0.41)***	2.28 (0.43)***	2.16 (0.33)#***
**Hemoglobin**	C-group	8.0 (0.6)	7.8 (0.6)##	7.9 (0.8)#	7.9 (0.9)###	7.7 (0.8) ###
(g/100 ml)	P-group	8.6 (0.6)	9.1 (0.6)**	9.2 (0.8)**	9.9 (0.6)***	10.1 (0.7)***
**pH**	C-group	7.50 (0.04)	7.50 (0.03)	7.51 (0.04)	7.50 (0.03)	7.51 (0.02)##
	P-group	7.52 (0.05)	7.50 (0.04)	7.49 (0.04)	7.45 (0.06)	7.43 (0.05)*
**BE**	C-group	7.74 (1.5)	8.08 (1.5)	8.31 (1.2)	8.49 (1.1)##	7.81 (1.1)##
(mmol/l)	P-group	7.65 (1.1)	7.36 (1.1)	6.85 (1.0)	5.68 (1.5)	5.29 (1.2)*
**P**_**a**_ **CO**_**2**_	C-group	5.3 (0.5)	5.5 (0.4)	5.3 (0.5)	5.5 (0.5)	5.2 (0.5)#
(kPa)	P-group	5.1 (0.7)	5.3 (0.6)	5.4 (0.4)	5.8 (0.9)	6.2 (0.7)
**HCO**_**3**_^**-**^	C-group	31.5 (1.4)	31.8 (1.4)	32.1 (1.1)	32.2 (1.0)#	31.6 (0.9)##
(mmol/l)	P-group	31.4 (0.9)	31.2 (1.1)	30.7 (1.0)	29.7 (1.7)	29.1 1.3)*
**P**_**a**_ **O**_**2**_	C-group	34.4 (11.9)	28.1 (5.4)	26.9 (4.0)	26.0 (4.8)	26.2 (5.6)
(kPa)	P-group	25.8 (4.9)	24.7 (4.0)	24.1 (5.5)	24.8 (4.5)	22.7 (5.3)
**TNF-α**	C-group	72.0 (28.9)	79.5 (27.3)	83.0 (27.0)	77.1 (24.1)	80.7 (29.0)
(pg/ml)	P-group	56.8 (21.1)	63.2(19.4)	64.7 (22.1)	87.8 (29.6)**	91.4 (38.4)*
**Interleukin-6**	C-group	3.6 (4.7)	9.0 (3.4)**	17.6 (3.4)***	23.9 (8.0)***	30.2 (14.5)**
(pg/ml)	P-group	10.5 (7.8)	16.5 (11.8)**	30.9 (13.1)***	52.8 (26.2)***	83.0 (64.4)

Results as means with SD in parentheses; Control group: C-group; Intervention group: P-group; *:P<0.05 **:P<0.01 ***:P<0.001; within group differences compared with baseline values; #:P<0.05 ##:P<0.01 ###:P<0.001; between group differences, same time.

Table 3 displays the blood glucose and markers of cerebral metabolism in fluid collected by cerebral microdialysis. Both study groups showed a reduction in blood glucose. A similar trend was seen in cerebral glucose. The other metabolic markers remained stable with no significant changes.

**Table 3:** **Markers of cerebral metabolism**

Parameter	Group	Baseline	60 min	120 min	180 min	240 min
**B-glucose**	C-group	6.6 (1.1)	6.4 (0.8)	6.2 (10.9)	5.6 (0.9)	4.9 (1.2)**
(mmol/l)	P-group	6.2 (2.2)	6.0 (2.0)	5.6 (1.7)	4.1 (2.1)	3.2 (1.3)**
**C-glucose**	C-group	2.5 (1.3)	2.6 (0.8)	2.1 (0.6)	1.7 (0.7)	1.4 (0.8)
(mmol/l)	P-group	3.2 (1.7)	3.1 (1.0)	2.7 (0.9)	2.1 (1.4)	1.7 (1.3)
**C-Lactate**	C-group	3.5 (0.9)	3.9 (1.0)	4.8 (1.4)	5.4 (1.7)	5.4 (1.7)
(mmol/l)	P-group	4.1 (1.6)	4.9 (1.7)	5.5 (2.3)	5.4 (2.7)	5.2 (2.6)
**C-Pyruvate**	C-Group	212 (137)	243 (103)	283 (110)	294 (117)	289 (91)
(μmol/l)	P-group	264 (135)	326 (127)	320 (116)	340 (168)	296 (140)
**L/P- ratio**	C-group	2.7 (3.4)	1.6 (1.1)	1.7 (1.3)	1.8 (1.5)	1.8 (1.5)
	P-group	1.1 (0.9)	1.1 (0.8)	1.2 (0.8)	1.1 (0.7)	1.2 (0.8)
**C-glycerol**	C-group	44.2 (29.1)	44.3 (17.7)	41.0 (12.2)	40.3(14.0)	38.5 (13.3)
(μmol/l)	P-group	45.8 (43.2)	39.5 (38.7)	45.5 (53.8)	47.0 (55.2)	58.8 (75.3)

*Values presented as mean with SD in parentheses; B: Blood; C: cerebral microdialysate; L/P-ratio: lactate/pyruvate ratio. Control group: C-group; Intervention group: P-group;*

**:P < 0.01; within group difference compared with baseline values.

Total water content in various tissue and organs are shown in Table 4. The kidneys and the lungs of the P-group animals contained significant less water than those of the C-group. Otherwise no significant differences in water content between the study groups were found.

**Table 4:** **Total tissue water content (TTW)**

Tissue	C-group	P-group	P
Right myocardium	4.15 (0.32)	4.04 (0.14)	NS
Left myocardium	3.98 (0.15)	3.87 (0.13)	NS
Lung	5.21 (0.25)	4.86 (0.19)	<0.01
Liver	3.47 (0.29)	3.66 (0.15)	NS
Right kidney	5.42 (0.75)	4.70 (0.22)	<0.05
Left kidney	5.36 (0.74)	4.55 (0.28)	<0.05
Stomach (muscularis)	4.79 (0.31)	4.50 (0.36)	NS
Stomach (mucosa)	4.77 (0.99)	4.08 (0.29)	NS
Pancreas	3.00 (0.53)	3.15 (0.23)	NS
Ileum (muscularis)	4.69 (0.59)	4.39 (0.34)	NS
Ileum (mucosa)	5.33 (0.27)	4.82 (0.82)	NS
Skeletal muscle	4.07 (0.40)	4.09 (0.32)	NS
Skin	2.84 (0.46)	2.68 (0.65)	NS
Brain	4.54 (0.27)	4.35 (0.32)	NS

*Results presented as mean with SD in parentheses. Control group: C-group; Intervention group: P-group.*

Table 5 demonstrates the microcirculatory changes as measured with colored microspheres. The perfusion of the ileal and ventricular mucosa was reduced in the P-group by the end of the experiment. Pancreas perfusion was reduced in the P-group and elevated in the C-group. After 240 min the values differed significantly between the groups (P < 0.001).

**Table 5:** **Microcirculatory flow**

	Baseline		240 min	
Tissue	C-group	P-group	C-group	P-group
Ileum muscularis	0.154 (0.192)	0.136 (0.152)	0.149 (0.210)	0.105 (0.122)
Ileum mucosa	0.373 (0.092)	0,462 (0.083)	0.372 (0.049)	0.364 (0.095)*
Corp.muscularis	0.053 (0,002)	0.055 (0.010)	0,065 (0,017)*	0,050 (0,015)
Corp.mucosa	0.350 (0,108)	0,352 (0.057)	0,396 (0,104)	0,291 (0,041)*
Pancreas	0.293 (0,048)	0.379 (0.11)	0.402 (0.060)*	0.255 (0.028)*###
Right kidney	2.385 (0.271)	3.136 (0.488)##	1.969 (0.384)*	1.506 (0.361)*
Left kidney	2.437 (0.187)	3.063 (0.484)##	2.006 (0.363)*	1.540 (0.397)*
Skin	0,014 (0,004)	0,016 (0,007)	0,018 (0,004)*	0,011 (0,003)#
Brain	0.366 (0.186)	0.386 (0.102)	0.354 (0.096)	0.489 (0.153)*
Right ventricle	0.511 (0.252)	0.654 (0.286)	0.583 (0.432)	0.633 (0.292)
Left ventricle	0.537 (0.274)	0.738 (0.331)	0.599 (0.441)	0.664 (0.342)
Muscle	0.021 (0.009)	0.022 (0.004)	0.021 (0.01)	0.018 (0.006)

*Microcirculatory flow in ml/min/100 g tissue; Results as means with SD in parentheses; Control group: C-group; Intervention group: P-group;*

*: P < 0.05; within group difference compared with baseline values;

*#: P < 0.05 ##: P < 0.01 ###: P < 0.001; between group difference, same time.*

The kidney perfusion was higher in the P-group than the C-group at baseline and decreased in both groups during the experiments. The percentage reduction was 19.8 (12.0) and 18.0 (7.7) in the left and right kidney of the C-group, and 50.1 (6.1) and 52.4 (5.0) in the left and right kidney of the P-group (P < 0.001, between group-difference). Skin perfusion increased in the C-group, and the levels were higher than in the P-group at the end of the experiments (P < 0.05). Cerebral perfusion was slightly increased in the P-group during the intervention (P < 0.05) with no between-group difference.

## Discussion

The clinical condition of acute intra-abdominal hypertension is frequently associated with elevated capillary permeability and abundant edema formation following local or systemic inflammatory processes [4, 15]. The present study demonstrate the reverse causal relationship: An acute elevation of intra-abdominal pressure contributes *per se* to a shift of fluid and proteins from the intravascular to the extravascular space leading to plasma volume contraction and deterioration of global perfusion. This constitutes a potential vicious circle in the development of IAH and organ failure, and has, to our knowledge, not previously been demonstrated in a large animal model.

In the present study both CVP_RA_ and CVP_FV_ were elevated in the P-group animals. Theoretically, capillary hydrostatic pressure (P_c_ ) depends on venous pressure (P_v_), venous resistance (R_v_) and blood flow (F) according to the following equation: P_c_ = P_v_ + F ∙ R_v_ [16]. Hence, the difference in fluid extravasation could be related to an increase of capillary hydrostatic pressure (P_c_) in the P-group.

Reduced lymphatic drainage from the abdominal cavity to the systemic circulation has been reported in dogs after elevation of intra-abdominal pressure to 20 mmHg [17]. This could also be a factor contributing to plasma volume contraction in IAH. Another potential mechanism could be increased capillary permeability due to ischemia and subsequent local inflammation. The present data does not support this hypothesis, however, since the levels of pro-inflammatory cytokines were low and did not differ between the groups.

In the current study, elevation of IAP caused a reduction in global perfusion as well as in the microcirculation of intestinal and ventricular mucosa, pancreas and the kidneys. This is in accordance with multiple animal studies and human case reports describing reduced intestinal perfusion due to elevated IAP [18–20]. On the other hand, cerebral perfusion increased slightly in the P-group, despite elevated intracranial pressure and a trend to reduction in cerebral perfusion pressure. The explanation could be an effect on cerebrovascular resistance caused by the slightly lower pH in the P-group. Previous experimental studies also reported raised intracranial pressure during conditions of intra-abdominal hypertension. A mechanism of compromised cerebral venous drainage has been suggested [21, 22]. Never the less, the stable levels of cerebral glycerol and lactate/pyruvate ratio in both study groups indicate adequate cerebral supply of oxygen and energy substrates during the experiments.

A few limitations of the study should be mentioned. The study is weakened by the postmortem data on water content in various tissue and organs where most samples showed no difference between the groups. The explanation is not clear, but could be related to the methods lack of sensitivity. Another explanation could be the fact that edema formation initially is located to tissues with high compliance, as for instance subcutaneous or retroperitoneal loose connective tissue [23]. Unfortunately, the range of samples did not include these. The present data are not able to determine the edema distribution in the experimental model and the issue should be addressed in a future study on IAH.

The data on kidney microcirculation surprisingly showed higher flow in the P-group than the C-group prior to intervention. Biologic variation and small numbers of animals in the study groups presumably could contribute to this. However, the percentage change after intervention demonstrated a significant difference between the groups, with a marked reduction in kidney perfusion seen in the P-group. This is consistent with data from other animal studies, suggesting that the mechanism of renal failure in IAH could be related to inadequate arterial blood flow and GFR [24, 25]. It underscores the fact that IAH is an independent risk factor for acute renal failure [4].

The experimental model does not quite mimic the clinical situation of intraabdominal hypertension, since no underlying pathology is present. The study was designed to explore the pathophysiological effects of elevated IAP on fluid shifts, independent of the underlying disorder. The findings may be relevant for the understanding of secondary ACS since intra-abdominal edema of various origins could be aggravated by the mechanism of a vicious circle. The results underline the importance of early interventions to prevent or treat acutely elevated intra-abdominal pressure.

Most experimental models on IAH have introduced carbon dioxide or saline into the abdominal cavity to raise IAP [26]. These models are limited by potential systemic effects following absorption of saline or by acid-base disorders following absorption of carbon dioxide [27]. In the present model, we applied insufflation of the inert gas helium, which has been associated with better cardiovascular stability in laparoscopic surgery when compared with CO_2_ [28].

Various methods for monitoring IAP have been evaluated in the literature and the measurement of urinary bladder pressure is advocated as the golden standard in the clinical routine [7]. In animal models CVP_FV_ has been shown to correlate well with changes in IAP. Our data are in line with this, showing that IAP and CVP_FV_ in the C-group corresponds and remained stable during the experiment, while in the P-group there was a significant, and parallel increase in IAP and CVP_FV._ Clinical studies, however, do not support the routine use of CVP_FV_ for monitoring IAP due to lack of accuracy in the lower pressure range [10, 29].

## Conclusion

In this porcine model of acute elevated intraabdominal pressure by insufflation of helium, the intervention was associated with increased extravasation of fluid and proteins, plasma volume reduction, reduced cardiac output and reduced perfusion of intraabdominal organs. The findings support the clinical recommendation of preventing IAH before hypo-perfusion and organ failure develops.

### Key message

Elevated intra-abdominal pressure is an independent cause of increased fluid extravasation rate and reduced plasma volume.

## Abbreviations

ACS: Abdominal compartment syndrome
CCO: Continuous cardiac output
CNS: Central nervous system
CO: Cardiac output
CPP: Cerebral perfusion pressure
CVP_FVP_: Central venous pressure of the femoral vein
CVP_RA_: Central venous pressure of the right atrium
FER: Fluid extravasation rate
Hb: Hemoglobin
Hct: Hematocrit
IAP: Intra-abdominal pressure
IAH: Intra-abdominal hypertension
ICP: Intra cerebral pressure
IL-6: Interleukin 6
I.V: Intravenous
KCl: Kalium chloride
KOH: Kalium hydroxide
MAP: Mean arterial pressure
MPAP: Mean pulmonary artery pressure
NFB: Net fluid balance
P_c_: Capillary hydrostatic pressure
PCWP: Pulmonary capillary wedge pressure
PV: Plasma volume
PVR: Pulmonary vascular resistance
SVR: Systemic vascular resistance
TNF-α: Tumor necrosis factor alpha
V_RBC_: Red blood cell volume.
